# Association of Mallampati scoring on airway outcomes in women undergoing general anesthesia with Supreme™ laryngeal mask airway in cesarean section

**DOI:** 10.1186/s12871-019-0796-5

**Published:** 2019-07-08

**Authors:** Hon Sen Tan, Shi Yang Li, Wei Yu Yao, Yong Jing Yuan, Rehena Sultana, Nian-Lin R. Han, Alex Tiong Heng Sia, Ban Leong Sng

**Affiliations:** 1 Department of Women’s Anaesthesia, KK Women’s and Children’s Hospital, 100 Bukit Timah Road, Singapore, Singapore; 2 Department of Anesthesiology and Perioperative Medicine, Quanzhou Macare Women’s Hospital, Quanzhou, Fujian Province China; 3 Department of Anesthesiology, Qinghai University Affiliated Hospital, Xining, Qinghar Province China; 4 Center for Quantitative Medicine, Duke-NUS Medical School, 8 College Road, Singapore, Singapore; 5 Division of Clinical Support Services, KK Women’s and Children’s Hospital, 100 Bukit Timah Road, Singapore, Singapore; 6 Anaesthesiology and Perioperative Sciences Academic Clinical Program, Duke-NUS Medical School, 8 College Road, Singapore, Singapore

**Keywords:** Laryngeal mask, Obstetrics, Cesarean section, Airway

## Abstract

**Background:**

Obstetric dfficult airway is a leading cause of maternal morbidity and mortality. The laryngeal mask airway (LMA) is often used as a rescue airway device after failed intubation, however, little is known about predictors of difficult LMA insertion, particularly in obstetrics. Since Mallampati scores of III/IV has been associated with difficult tracheal intubation, our present study aims to investigate if Mallampati score (MP) could predict airway outcomes for LMA use in obstetrics.

**Methods:**

This prospective cohort study was performed at a single-center: Quanzhou Women’s and Children’s Hospital, Fujian Province, China. Five hundred and eighty-four parturients undergoing elective cesarean section under general anesthesia were recruited. The primary outcome was time to effective ventilation, and secondary outcomes included first attempt insertion success, seal pressure, ventilation and hemodynamic parameters, occurrence of clinical aspiration, and maternal and fetal outcomes.

**Results:**

The parturients were classified into two groups based on MP of III/IV (High MP: 61) versus I/II (Low MP: 523). BMI was higher in the High MP group than in the Low MP group (mean (SD) 29.3 (7.0) vs 26.8 (3.1), *p* <  0.0001). There was no difference in maternal age, ASA status and gestational age. There was similar time to effective ventilation (mean (SD) High MP: 14.9 (4.5) vs Low MP: 15.7 (4.4) seconds, *p* = 0.2172), and first attempt success rate, seal pressure, and peak airway pressure. No clinical aspiration was noted. The incidence of blood on SLMA was higher in the High MP group than in Low MP (4 (6.6%) vs 4 (0.8%), *p* = 0.001). There was no difference in sore throat, voice hoarseness, maternal satisfaction and fetal outcomes.

**Conclusion:**

High MP was not associated with reduced SLMA airway outcomes in cesarean section under general anesthesia, but may increase the risk of blood found on SLMA upon removal.

**Trial registration:**

This study was registered at http://www.clinicaltrials.gov, identifier: NCT02026882, retrospectively registered. Date of registration: December 31, 2013.

## Background

Airway complication is one of the main causes of anesthesia-related obstetric adverse events. There are several complex clinical challenges faced in managing the parturient including the risk of pulmonary aspiration, physiological changes of pregnancy, urgent nature of cesarean section, and balancing the potentially conflicting needs of the mother and fetus. Endotracheal intubation has long been considered standard of care for obstetric airway management [1, 2], but comes with the concomitant risk of difficult intubation; a potentially life-threatening situation. The Mallampati score and other bedside airway assessments have been used to predict difficult intubation in adult patients. A recent Cochrane systematic review reported the specificity and sensitivity of the modified Mallampati score in diagnosing difficult tracheal intubation as 0.87 and 0.51, respectively [3].

With the additional risk of failed intubation posed by the obstetric airway (estimated at 0.4%), the laryngeal mask airway (LMA) has emerged as a second-line airway device [4–8]. Additionally, the viability of second-generation LMAs with a double-lumen specifically designed to physically isolate the alimentary tract and reduce the risk of aspiration [9–12] was highlighted by its inclusion in the obstetric-specific guideline jointly released by Obstetric Anaesthetists’ Association (OAA) and Difficult Airway Society (DAS) [13]. Our group has also demonstrated the efficacy of LMA use for obstetric airway management in several prospective cohort studies [12, 14].

Notwithstanding its utility as a rescue airway device, we should be cognizant that the incidence of first-attempt LMA failure in obstetrics is approximately 2% [12, 14]. Additionally, the efficacy of bedside tests to predict LMA failure is poorly elucidated in the general population, with a paucity of obstetric-specific data. Despite its widespread use in predicting difficult intubations, the modified Mallampati score in non-obstetric cases has been shown to predict LMA failure by some authors [15], whereas others reported otherwise [16, 17]. This gap in knowledge, especially relating to parturients, is concerning, as the ability to accurately and rapidly predict LMA failure a priori is essential in obstetrics, due to the reliance on the LMA as a rescue airway device, as well as the limited time available before the risk of maternal or fetal morbidity increases.

Therefore, it is vital to predict when LMA failure is likely to occur, and formulate an alternate airway plan. Additionally, to be an effective rescue airway the factors associated with LMA failure could be different from those predicting failure to intubate. This prospective cohort study aims to investigate the association between the modified Mallampati score and airway outcomes of Supreme™ LMA insertion for cesarean section under general anesthesia.

## Methods

This is a secondary analysis of a previously-published prospective cohort study [14], which was approved by the Quanzhou Women’s and Children’s Hospital Institutional Review Board (dated 11 November 2013) and registered at Clinicaltrials.gov registry (NCT02026882). The study was conducted at Quanzhou Women’s and Children’s Hospital, Fujian Province, China, and patient recruitment occurred between December 2013 and November 2014. Largely due to patient preference, approximately 35% of parturients undergo cesarean section at the study center, with the majority performed under general anesthesia using the Supreme™ LMA (SLMA). Hence, the SLMA has been established as part of the routine airway management at the study center.

Parturients who were healthy or with well-controlled medical conditions (American Society of Anesthesiologists (ASA) physical status classification II) undergoing elective cesarean section under general anesthesia were recruited. We excluded patients with BMI > 35 kg/m^2^, upper respiratory tract or neck pathology, or self-reported reflux disease. After fasting for a minimum of 4 h, the modified Mallampati score was determined by an independent assessor and the patients divided into two groups: Mallampati III/IV (High MP) and Mallampati I/II (Low MP).

The induction of anesthesia and SLMA insertion reflects the clinical practice at the study center, as well as the cohort study that forms the basis for this secondary analysis [14]. As per institutional standard, all parturients were premedicated with intravenous ranitidine, and monitored with electrocardiogram, pulse oximetry, capnography and non-invasive blood pressure monitor. The chosen SLMA size was based on the manufacturer’s guidelines and the discretion of the attending anesthesiologist. All SLMA were inserted by three investigators (Yao, Li, and Yuan), each with at least five years of experience in its use for cesarean delivery. Anesthesia was commenced via rapid sequence induction with the application of cricoid pressure by a trained anesthetic assistant, and administration of propofol and succinylcholine, followed by SLMA insertion using the recommended rotational technique. After cuff inflation to 60 cmH_2_O via a manometer, assessment of successful SLMA placement was made by auscultation of breath sounds and the capnographic evidence of end-tidal carbon dioxide, and cricoid pressure was released. The use of additional maneuvers such as chin lift, jaw thrust, or head tilt were permitted. Rocuronium was used to maintain intraoperative muscle relaxation, and fentanyl was administered for analgesia after delivery of the fetus.

We recorded (1) the number of insertion attempts required, with each attempt defined as insertion and complete removal of the SLMA; and (2) the time to effective airway placement, from when the SLMA was picked up until the first end-tidal carbon dioxide capnogram appeared. Subsequently, a pre-mounted #14 orogastric tube was inserted through the gastric drainage port, and placement confirmed by aspiration of gastric contents and auscultation of a “swoosh” over the epigastrium with air injection, followed by suctioning of the orogastric tube before surgery commenced. Finally, oropharyngeal leak pressure was measured by shutting the adjustable pressure-limiting valve and maintaining 3 L/min fresh gas flow into the closed circuit. Cesarean section was allowed to commence if the following were met: presence of square-wave capnogram, cuff pressure of 60 cmH_2_O, positioning of the SLMA bite block between the incisors, successful orogastric tube insertion, and leak pressure > 20cmH_2_O. If successful SLMA insertion was not accomplished (1) after two attempts, (2) within one minute, or (3) before desaturation ensued, endotracheal intubation would be performed.

Perioperative anesthesia management during the original cohort study [14] reflects the clinical practice at the study center. With all parturients positioned left lateral tilt using a wedge, anesthesia was maintained with 1.5 to 2% sevoflurane and 50:50 mix of nitrous oxide in oxygen. We recorded the incidence of airway complications including airway obstruction, inadequate oxygenation or ventilation, and the presence of signs of clinical aspiration such as hypoxemia, auscultation of wheezing or crepitations, and postoperative dyspnea. Parturients were ventilated with tidal volume of 6 to 10 ml/kg, and respiratory rate of 10 to 16 breaths/min. The obstetricians were advised to avoid excessive fundal pressure during fetal delivery. Upon completion of surgery, suctioning and removal of the orogastric tube was performed, and the SLMA was withdrawn and inspected for blood. An independent assessor determined the incidence of sore throat and voice hoarseness prior to discharge from the post anesthesia care unit.

Our primary outcome was time to effective ventilation, as defined above. Secondary outcomes included the rate of successful first-attempt SLMA insertion, oropharyngeal leak pressure, ventilation parameters such as tidal volume and respiratory rate, hemodynamic parameters including heart rate and blood pressure at 2 and 5 min after induction, amount and pH of gastric aspirate, the pH of the SLMA laryngeal surface, and the presence of clinical aspiration. Obstetric and fetal outcomes included neonatal weight, Apgar scores, and umbilical venous pH.

### Statistical analysis

All demographic, anesthetic, and clinical categorical data were summarized as frequency with corresponding proportion, and continuous variables were expressed as mean (standard deviation (SD)) or median [interquartile range (IQR)], whichever appropriate. The difference between categorical data was tested using the Chi-Square test, while Student’s t-test and Mann-Whitney U test were used for parametric and non-parametric continuous data, respectively. Simple linear and multivariable linear regression methods were utilized to determine associated risk factors for our primary outcome of time to effective ventilation. Associations identified from linear regression were expressed as estimate (β) with 95% confidence interval (95%CI). Significance level was set at *p* <  0.05 and all tests were two-sided. Data analysis was generated using SAS 9.3 software (SAS Institute Inc., Cary, NC, USA).

We performed a sample size calculation based on the following assumptions: a meaningful clinical difference of at least 10 s in time to effective ventilation between “High MP” and “Low MP” groups, pooled SD of 4.5, allocation ratio of 1:8, use of two-sample independent t-test, level of significance of 5% and > 95% power. The study was adequately powered (> 95%) with 584 parturients.

## Results

We recruited 584 parturients. Of these, 61 were found to have Mallampati III/IV (High MP) and 523 with Mallampati I/II (Low MP) (Table 1). There was no withdrawal or dropout. Demographic and clinical characteristics are summarized in Table 2. The BMI in the High MP group was higher than in the Low MP group (mean (SD) 29.3 (7.1) vs 26.8 (3.1), *p* = 0.0001), but there was no significant difference in maternal age, fetal weight, or gestational age. The duration of surgery was similar, with mean (SD) 31.0 (11.1) minutes in the High MP group vs 29.3 (9.2) minutes in the Low MP group (*p* = 0.4159).

**Table 1:** Distribution of modified Mallampati score among participants undergoing cesarean section under general anesthesia

	Frequency, *n* = 584	Proportion (%)
Mallampati I	226	38.70
Mallampati II	297	50.86
Mallampati III	56	9.59
Mallampati IV	5	0.86

**Table 2:** Baseline patient demographics and clinical characteristics of participants with either modified Mallampati scores of III/IV versus I/II undergoing cesarean section under general anesthesia

	Mallampati Score		*p*-value
	III to IV	I to II	
Age (years), mean (SD)	28.8 (5.4)	28.9 (4.0)	0.5315
BMI (kg/m^2^), mean (SD)	29.3 (7.1)	26.8 (3.1)	**0.0001**
Duration of surgery (min), mean (SD)	31.0 (11.1)	29.3 (9.2)	0.4159
Gestation (weeks), median [IQR]	38.0 [37.1 to 39.0]	38.3 [37.1 to 39.0]	0.1024
Fetal weight (g), mean (SD)	2995 (580)	3017 (532)	0.3608

Values are presented as mean (SD) or median [IQR]

Significance level was set at *P* < 0.05

Anesthetic outcomes are presented in Table 3. There was no significant difference in our primary outcome of time to effective ventilation, with 14.9 (4.5) seconds for the High MP group versus 15.7 (4.4) for the Low MP group (*p* = 0.2172). Similarly, there was no difference in first attempt insertion success rate of 98.4% for the High MP group versus 98.3% for the Low MP group. The lowest SpO_2_, seal pressure and peak airway pressures were similar between the groups. No episodes of hypoxemia, laryngospasm, or bronchospasm were observed intra-operatively.

**Table 3:** Anesthetic outcomes of participants with either modified Mallampati scores of III/IV versus I/II while undergoing cesarean section under general anesthesia

	Mallampati Score		*p*-value
	III to IV	I to II	
Time to effective ventilation (s), mean (SD)	14.9 (4.5)	15.7 (4.4)	0.2172
No. of insertion attempts of LMA > 1, n (%)	1 (1.6%)	9 (1.7%)	0.6373
No. of insertion attempts of gastric tube, median [IQR]	1 [1–1]	1 [1–1]	1.0000
Seal pressure (cmH2O), median [IQR]	27 [26–31]	27 [25–30]	0.5811
Peak airway pressure (cmH2O), median [IQR]	18 [16–23]	18 [15–21]	0.1134
Lowest SpO_2_ (%), median [IQR]	99 [98–99]	99 [98–99]	0.5812
Baseline systolic blood pressure (mmHg), mean (SD)	128.8 (15.7)	121.8 (12.4)	**0.0001**
Systolic blood pressure 2 min after induction (mmHg), mean (SD)	126.9 (21.2)	115.9 (17.3)	**< 0.0001**
Systolic blood pressure 5 min after induction (mmHg), mean (SD)	111.9 (13.4)	108.5 (14.6)	0.0802
Baseline heart rate (beats/min), mean (SD)	89.0 (12.0)	87.4 (11.3)	0.2866
Heart rate 2 min after induction (beats/min), mean (SD)	94.0 (15.4)	94.4 (17.2)	0.8725
Heart rate 5 min after induction (beats/min), mean (SD)	86.1 (13.1)	88.4 (18.2)	0.3481

Values are presented as mean (SD), n(%) or median [IQR]

Significance level was set at *P* < 0.05

Maternal and fetal outcomes are summarized in Table 4. There was no clinical aspiration noted, and the volume of gastric aspirate was similar between the groups. However, the pH of the SLMA laryngeal surface was statistically lower in the Low MP group: 7.2 (0.4) for High MP versus 7.0 (0.5) for Low MP (*p* = 0.0191). The presence of blood on the SLMA was more frequently noted in the High MP group with 4 (6.6%) versus 4 (0.8%) in the Low MP group (*p* = 0.0010), but there were no significant differences in sore throat (*p* = 0.6071), voice hoarseness (*p* = 0.1585), or maternal satisfaction (*p* = 0.5131). Fetal outcomes, including Apgar scores at 1 and 5 min, and venous cord pH were similar between the groups.

**Table 4:** Maternal and fetal outcomes of participants with either modified Mallampati scores of III/IV versus I/II undergoing cesarean section under general anesthesia

	Mallampati Score		*p*-value
	III to IV	I to II	
Gastric aspirate volume (mL), mean (SD)	18.3 (27.9)	13.7 (11.7)	0.4256
pH of gastric aspirate, mean (SD)	2.6 (1.0)	2.3 (0.8)	**0.0314**
pH of SLMA laryngeal surface, mean (SD)	7.2 (0.4)	7.0 (0.5)	**0.0191**
Blood on SLMA, n (%)	4 (6.6%)	4 (0.8%)	**0.0010**
Sore throat, n (%)	4 (6.6%)	34 (6.5%)	0.6071
Voice hoarseness, n (%)	0	4 (0.8%)	0.1585
Maternal satisfaction (%), mean (SD)	86.8 (9.2)	86.0 (8.5)	0.5131
Fetal Apgar at 1 min, mean (SD)	9.2 (1.2)	9.2 (1.1)	0.8250
Fetal Apgar at 5 min, mean (SD)	9.6 (0.9)	9.7 (0.7)	0.5198
Venous cord pH, mean (SD)	7.3 (0.1)	7.3 (0.1)	0.4969

Values are presented as mean (SD) or n (%)

Significance level was set at *P* < 0.05

Univariate linear regression method showed that age and baseline systolic blood pressure were significantly associated with time to effective ventilation (Table 5); while multivariable linear regression showed that age (β (95%CI): 0.012 (0.007, 0.017)) and baseline systolic blood pressure (β (95%CI): − 0.004 (− 0.006, − 0.002)) were significantly associated with time to effective ventilation (Table 5).

**Table 5:** Univariate and multivariable linear regression to find associated risk factors for time to effective ventilation

Variables	Univariate linear regression		Multivariable linear regression	
	Estimate (95%CI)	*P* - value	Estimate (95%CI)	*P* - value
Age (years)	0.17 (0.08, 0.25)	**0.0001**	0.012 (0.007, 0.017)	**< 0.0001**
BMI (kg/m^2^)	0.02 (−0.07, 0.12)	0.6074		
Gestation (weeks)	0.19 (0, 0.38)	0.0537		
Fetal weight (g)	0.0004 (−0.0003, 0.0011)	0.2252		
Baseline systolic blood pressure (mmHg)	−0.05 (− 0.08, − 0.03)	**0.0002**	−0.004 (− 0.006, − 0.002)	**< 0.0001**
Heart rate (bpm)	0.003 (− 0.029, 0.035)	0.8514		

Significance level was set at *P* < 0.05

## Discussion

This prospective cohort study of 584 parturients undergoing cesarean section under general anesthesia compared the airway outcomes of the SLMA in patients with lower Mallampati scores (I or II) versus higher scores (III or IV). Out of 584 patients, 61 (10.4%) had Mallampati scores of III or IV. We found no association between Mallampati scores and time to effective ventilation, first attempt success rate, or SLMA seal pressure. There was no clinical aspiration detected, and the pH of the SLMA laryngeal surface was not indicative of gastric regurgitation. However, higher Mallampati scores was associated with increased risk of blood found on the SLMA, which may indicate increased risk of oropharyngeal trauma on insertion, but without corresponding change in sore throat, voice hoarseness, or maternal satisfaction.

Our results suggest that LMA outcomes in obstetric general anesthesia was not significantly affected by higher Mallampati scores. The Mallampati score is postulated to be related to the size of the tongue base during difficult laryngoscopy due to occlusion of the view of pharyngeal structures [15]. This is supported by Cormack and Lehane, who cited large or poorly mobile tongue and anterior larynx as potential causes of difficult laryngoscopy [18].

Although previous studies associated higher Mallampati scores with increased LMA failure rates [15], our findings concur with studies demonstrating poor relation between Mallampati score and LMA outcomes [16, 17]. Furthermore, the time to effective ventilation and first attempt SLMA insertion success rate reported here is comparable to other LMA studies in obstetric general anesthesia [12], despite application of cricoid pressure which may impede LMA insertion into the post-cricoid hypopharyngeal space. This excellent success rate despite high Mallampati scores may be stem from the postulation by Brain that intubation difficulties were associated with anteriorly sited larynx and therefore predicted by higher Mallampati scores, whereas a posteriorly sited larynx may increase LMA failure by blocking downward progress of the LMA tip [19]. Other attenuating factors include (1) routine use of SLMA for cesarean delivery at the study center, (2) insertion by experienced anesthetists, and (3) the relative rigidity of the SLMA.

A common concern of LMA use in obstetrics relates to the inherently higher risk of gastric regurgitation and pulmonary aspiration, exacerbated by concomitant obesity, labor pain, or opioid analgesia. In this study, we did not detect any clinical aspiration or regurgitation, likely due to (1) the ability of second-generation LMAs to isolate the alimentary tract and facilitate gastric decompression, (2) better pharyngeal seal that prevents stomach insufflation and regurgitated gastric contents from entering the airway, (3) use of rapid-sequence induction and cricoid pressure, and (4) careful patient selection with exclusion of patients at high risk for regurgitation. However, this study was not powered to investigate the risk of pulmonary aspiration.

There are several limitations in this study. First, we are cognizant that bedside tests, including the Mallampati score, are considered by some to be poor predictors of difficult intubation especially if used in isolation [20, 21]. However, a recent meta-analysis by Lee et al. reported a five-fold increase in accuracy of the modified Mallampati score for predicting difficult laryngoscopy in obstetric patients [20], thus lending weight to its use in parturients. Additionally, we investigated the Mallampati score independently instead of in combination with other bedside tests for difficult intubation due to scarce data of the utility of such tests in obstetrics, and concerns that a possible correlation with SLMA failure may be hidden if several predictive factors were summated.

Second, we utilized clinical markers of SLMA airway outcomes, such as time to ventilation, first attempt insertion success rate, and seal pressure, in contrast to other studies that assessed LMA position via a fiberoptic grading scale [17]. Several studies utilizing fibreoptic grading have reported a 50% incidence of suboptimal anatomical positioning, allegedly resulting in gastric air insufflation [17, 22]. While we agree that fiberoptic grading would provide additional information on SLMA placement, our routine practice does not include fiberoptic assessment, and this study was a pragmatic evaluation of the use SLMA in obstetrics, hence our focus was on its ability to achieve adequate ventilation. Furthermore, gastric insufflation may be attenuated by our reported average seal pressure being 9cmH_2_O greater than the average peak airway pressure regardless of Mallampati score, as well as gastric decompression via an orogastric tube.

Third, we deliberately excluded patients at high risk of gastric regurgitation, including parturients with BMI of 35 kg/m^2^ or higher. The retrospective study by Ramachandran et al. reported that elevated BMI of 29.3 versus 26.9 was associated with LMA Unique failure, but Mallampati scores of III to IV were not [23]. Despite the “High MP” group having significantly higher mean BMI of 29.3 versus 26.8 kg/m^2^ in the “Low MP” group, we did not find any corresponding increased SLMA failure, although we were not specifically assessing the effects of increasing BMI on SLMA outcomes. Finally, we did not directly correlate failed intubation with LMA failure, and therefore cannot comment on the use of SLMA as a rescue airway.

## Conclusion

Our prospective cohort study suggests that higher Mallampati score is not associated with longer time to effective ventilation when using the Supreme LMA in parturients undergoing elective cesarean delivery under general anesthesia, with similar time to effective ventilation, first attempt SLMA success rate, and seal pressure. More work is required to evaluate the accuracy of other bedside tests on predicting LMA airway outcomes in obstetrics.

## Data Availability

The datasets generated and analyzed for this manuscript are not publicly available, but could be obtained from the corresponding author on reasonable request.

## Abbreviations

ASA: American Society of Anesthesiologists
BMI: Body mass index
DAS: Difficult Airway Society
LMA: Laryngeal mask airway
MP: Mallampati Score
OAA: Obstetric Anaesthetists’ Association
SD: Standard deviation
SLMA: Supreme™ LMA
